# Novel genetic susceptibility loci identified by family based whole exome sequencing in Han Chinese schizophrenia patients

**DOI:** 10.1038/s41398-020-0708-y

**Published:** 2020-01-16

**Authors:** Mo Li, Lu Shen, Luan Chen, Cong Huai, Hailiang Huang, Xi Wu, Chao Yang, Jingsong Ma, Wei Zhou, Huihui Du, Lingzi Fan, Lin He, Chunling Wan, Shengying Qin

**Affiliations:** 1 Bio-X Institutes, Key Laboratory for the Genetics of Developmental and Neuropsychiatric Disorders (Ministry of Education), Shanghai Jiao Tong University, Shanghai, China; 2 Analytic and Translational Genetics Unit, Department of Medicine, Massachusetts General Hospital and Harvard Medical School, Boston, MA USA; 3 Broad Institute of Harvard and MIT, Cambridge, MA USA; 4 Psychiatric Hospital of Zhumadian City, Henan, China; 5 The Third Affiliated Hospital, Guangzhou Medical University, Guangdong, China; 6 Collaborative Innovation Center, Jining Medical University, Shandong, China

**Keywords:** Diagnostic markers, Clinical genetics

## Abstract

Schizophrenia (SCZ) is a highly heritable psychiatric disorder that affects approximately 1% of population around the world. However, early relevant studies did not reach clear conclusions of the genetic mechanisms of SCZ, suggesting that additional susceptibility loci that exert significant influence on SCZ are yet to be revealed. So, in order to identify novel susceptibility genes that account for the genetic risk of SCZ, we performed a systematic family-based study using whole exome sequencing (WES) in 65 Han Chinese families. The analysis of 51 SCZ trios with both unaffected parents identified 22 exonic and 1 splice-site de novo mutations (DNMs) on a total of 23 genes, and showed that 12 genes carried rare protein-altering compound heterozygous mutations in more than one trio. In addition, we identified 26 exonic or splice-site single nucleotide polymorphisms (SNPs) on 18 genes with nominal significance (*P* < 5 × 10^−4^) using a transmission disequilibrium test (TDT) in all the families. Moreover, TDT result confirmed a SCZ susceptibility locus on 3p21.1, encompassing the multigenetic region *NEK4-ITIH1-ITIH3-ITIH4*. Through several different strategies to predict the potential pathogenic genes in silico, we revealed 4 previous discovered susceptibility genes (*TSNARE1*, *PBRM1*, *STAB1* and *OLIG2*) and 4 novel susceptibility loci (*PSEN1*, *TLR5*, *MGAT5B* and *SSPO*) in Han Chinese SCZ patients. In summary, we identified a list of putative candidate genes for SCZ using a family-based WES approach, thus improving our understanding of the pathology of SCZ and providing critical clues to future functional validation.

## Introduction

Schizophrenia (SCZ) is a severe psychiatric disorder that has a global prevalence of approximately 1%^1^. The hallmark of SCZ is characterized by positive symptoms (i.e., hallucinations, delusions and disorganization) and negative symptoms (i.e., impaired motivation, reduced spontaneous speech and social withdrawal). Over decades, mounting evidences have strongly suggested that SCZ has a significant genetic component, indicated by its heritability estimates of 60–80%^2,3^. Currently, SCZ has been widely regarded as a highly complex genetic disorder that is caused by multiple genetic variants in cooperation with environmental risk factors^4^. In addition, common variants only confer a relatively small influence on SCZ risk^5^ and affect highly heterogeneous in different populations^6^. Therefore, an appropriate approach that accounts for the high heritability and strong familial aggregation of SCZ is essential to study the genetic predisposition to SCZ^7^.

Many approaches have been applied to study the genetic factors that are associated with susceptibility to SCZ, including linkage analysis, genome wide association study (GWAS), copy number variant (CNV) analysis strategy and whole exome sequencing (WES)^8^. In the past several years, a number of GWASs have identified over 100 genetic loci associated with SCZ risk^5,9–16^. Such studies to detect susceptibility genes require a very large sample size since the influences of individual susceptibility variants on SCZ risk may be small^17^. In addition, despite specific rare CNVs are now recognized as established risk factors for SCZ susceptibility^18–26^, these rare CNVs are usually potent risk factors of several other different psychiatric disorders and they often involve with many genes^7^. Recently, de novo mutations (DNMs) have been shown to be a highly successful new approach to study SCZ risk using family-based WES^27–34^. These results give us new options to explore the possible pathogeneses of SCZ.

Unlike traditional case-control studies, family-based studies can exclude some confounding factors, such as fractional environmental factors bias and population structure difference. More important, family-based studies allow for directly observation of DNMs, compound heterozygous mutations and transmission disequilibrium mutations in probands. In particular, specific compound heterozygosity has been demonstrated to underlie autism spectrum disorders^35,36^. However, the roles of these mutations in other mental disease are still not completely understood.

**Fig. 1:**
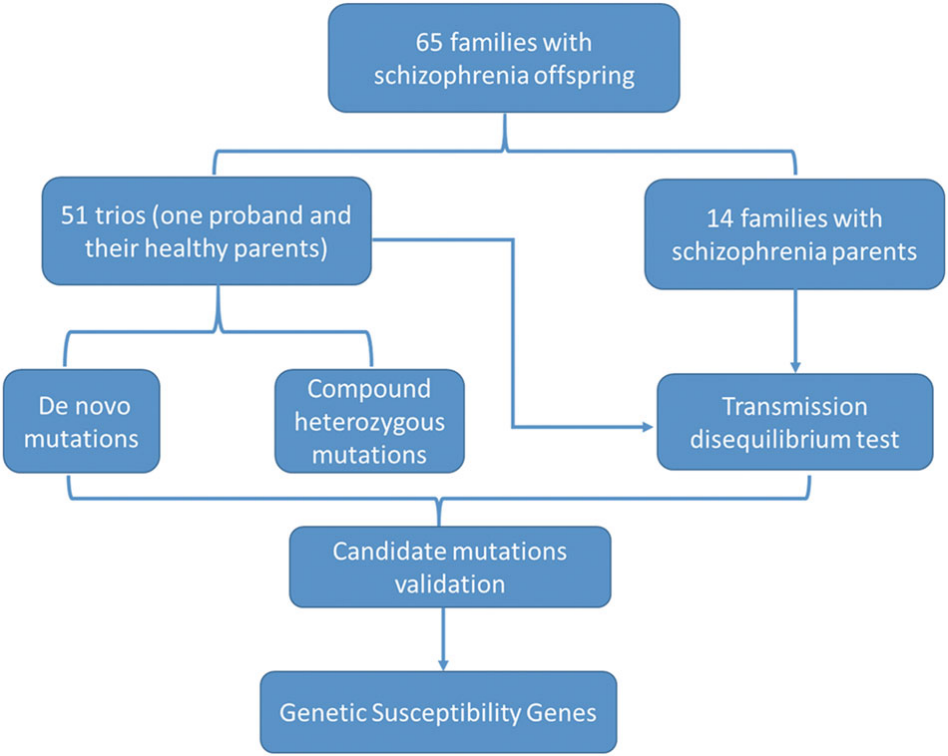
Study design. Our study design consisted of looking for novel susceptibility genes that account for the genetic risk of schizophrenia through a whole-exome sequencing method. First, we recruited 65 schizophrenia families. In our recruited samples, there were 51 trios (one affected proband and their healthy parents), 13 families with one schizophrenic parent and 1 family with both schizophrenic parents. Next, we used multiple strategies to identify variants that are associated with susceptibility to SCZ including de novo mutation, compound heterozygous mutation and transmission disequilibrium test. Then, we performed validation study for each class of variants. Finally, confirmed a list of putative candidate genes that were found to be associated with schizophrenia.

In this study, we implemented family-based WES analysis in 65 Han Chinese families with one SCZ offspring to identify DNM or inherited variants that are associated with susceptibility to SCZ. The overall experimental design is presented in Fig. 1. Our findings will help to further understand the etiology of SCZ and elucidate the biological mechanism underlying the familial liability of SCZ.

## Materials and methods

### Subjects

A total of 65 SCZ offsprings (32 males and 33 females, aged 46.3 ± 8.6 years) and their biological parents were recruited from psychiatric hospitals in Shanghai, China. We ascertained the diagnosis based on DSM-IV criteria and consensus clinical judgments of two fully qualified senior psychiatric physicians. In our recruited samples, there were 51 SCZ trios (one affected proband and their healthy parents), 13 families with one schizophrenic parent and 1 family with both schizophrenic parents. All subjects in study were of Han Chinese origin.

This study was approved by the Ethical Committee of Bio-X Institutes of Shanghai Jiao Tong University. All subjects or their legal guardians provided written informed consent to their participation before enrollment of this study in accordance with the guidelines laid out in the Declaration of Helsinki^37^.

### DNA extraction and WES

Genomic DNA was extracted from peripheral blood using the QIAamp DNA Mini Kit (QIAGEN GmbH, Hilden, Germany). Whole-exome capture library was performed using Agilent SureSelect^XT^ Target Enrichment System Human All Exon V5 + UTRs (Agilent Technologies, Santa Clara, CA, USA) according to the manufacture’s protocols. After that, libraries were assessed for sequencing using Agilent 2100 Bioanalyzer High Sensitivity DNA chip (Agilent Technologies, Santa Clara, CA, USA). Next, trusted high-level libraries were sequenced on Illumina Hiseq 2500 (Illumina, San Diego, CA, USA).

The raw sequencing reads (in fastq format) were mapped to the human reference genome (hg19) with the Burrows-Wheeler Aligner (BWA, version 0.7.15, http://bio-bwa.sourceforge.net/) tool. Then, the mapped reads were processed by Picard (version 2.15.0, https://github.com/broadinstitute/picard) to detect the polymerase chain reaction (PCR) duplicates. After marking the duplicates, Genome Analysis Toolkit (GATK, version 3.8, https://software.broadinstitute.org/gatk/) was then used to perform local realignment and base quality score recalibration (BQSR) to realign insertion-deletions (indels) and correct for the base quality scores from BAM files. GATK *HaplotypeCaller* tool was used to call the variants. The called variants (in VCF formats, version 4.0) file of every sample were merged using *GenotypeGVCFs* and then filtered using *VariantRecalibrator* tool in GATK.

### Identification of DNMs and compound heterozygous mutations

The 51 SCZ trios with both healthy parents were used to identify DNMs since DNMs are more likely to account for sporadic forms of the disease^29^. Identification of DNM candidates was performed using variant annotation module (*PossibleDeNovo*) in GATK. In addition, all the detected DNMs must not been described in any reported database. The exonic or splice site DNMs with high confidence were considered as candidates for subsequent validation and analysis.

Compound heterozygosity was defined as an individual carrying two heterozygous genotypes from one maternal and one paternal copy of different locations within the same gene^38^. We follow this definition to screen protein-altering (including nonsynonymous and stop loss/gain) compound heterozygous mutations in the 51 trios. Heterozygous mutations that had minor allele frequency (MAF) < 0.01 in East Asian population in 1000 Genomes Project database (https://www.internationalgenome.org/) and their hit genes appeared in more than one trio were selected as heterozygous mutations candidates.

ANNOVAR software^39^ was used to annotate all the DNMs and compound heterozygous mutations. The annotations included the predicted functional consequence according to RefSeq transcripts, whether they overlapped a segmental duplication, predicted effect on protein function and corresponding allele frequencies in multiple public databases (such as: 1000 g, esp6500, ExAC).

### Validation of DNMs and compound heterozygous mutations

The selected candidate DNMs and compound heterozygous mutations as described below were validated by Sanger sequencing. Primers were designed using DNAStar (https://www.dnastar.com/) software. PCR was performed using the AmpliTaq Gold DNA Polymerase (Applied Biosystems, Foster City, CA, USA) following the manufacturer’s instructions. The products of PCR were then sequenced using a 3730XL DNAnalyzer (Applied Biosystems, Carlsbad, CA, USA) and Mutation Surveyor software^40^ was used for mutation detection analysis.

### Variants quality control and family-based association analysis

Transmission disequilibrium test (TDT) was performed to test the family-based association between common single nucleotide polymorphisms (SNPs) and SCZ susceptibility. Quality control (QC) filters were applied to all the 65 family members. For data cleaning, systematic stepwise quality filtering of raw genotyping data was done using PLINK software (version 1.9, http://zzz.bwh.harvard.edu/plink/plink2.shtml). Samples with call rate less than 95% and high heterozygosity rate (deviation from triple standard deviations) were removed from further study. In addition, we validated the sex of each individual by concordance between self-reported sex and sex determined by genotyping. Principal component analysis (PCA) was conducted based on the passed QC variants using PLINK software. SNPs with a call rates less than 95%, difference in missing genotype rate between cases and controls greater than 5%, minor allele frequency below 0.01 or significant deviation from expected Hardy-Weinberg equilibrium (*P* < 1.0 × 10^−6^) were removed from further family-based association analysis.

### In silico analysis of candidate genes

We used several different strategies to predict the potential pathogenic genes in SCZ. First, we explored whether the candidate genes had been previously identified in other independent GWAS or genetic association studies on SCZ. Then, a residual variation intolerance score (RVIS, version 4, http://genic-intolerance.org/) analysis was then performed to assess the intolerance of variations in the candidate genes, therefore inferring their pathogenic impacts in SCZ. RVIS was a statistics that rank the genes of interest according to their tolerance to functional genetic variation by comparing the number of observed variants in the gene to the genome wide expectation given the number of neutral variation the gene has. At last, we systematically investigated the networks of functional interactions among the candidate genes using a web-based tool called Association Network Integration Algorithm (GeneMANIA) (http://genemania.org/). The results were visualized in Cytoscape (version 3.5.1, https://cytoscape.org/). The hub genes were defined as: the top 3 genes that appeared most according all the 12 cytoHubba ranking methods using Cytoscape software. Subsequently, the Molecular Complex Detection (MCODE) algorithm in Cytoscape software was used to screen modules. An MCODE score > 3 and a node number > 3 were taken as the criteria to define a core module.

### Statistical analysis

TDT was performed to test the family-based single maker association using PLINK software. In order to obtain the candidate variant associations, Pearson chi-square test was performed to evaluate significance of genotype frequency distributions. The power and pre-specified effect size was calculated by G-power software (https://stats.idre.ucla.edu/other/gpower/). R package (qqman, https://cran.r-project.org/web/packages/qqman/) and Haploview tool (https://www.broadinstitute.org/haploview/) was used to visualize TDT results using Q-Q and Manhattan plot respectively. The linkage disequilibrium analysis of significant SNPs locating at 3q21.1 was through Haploview tool.

## Results

### Summary of exome sequencing data

Among all subjects, an average of ~72.2 million reads was available with high enough depth (average, 47.8×). The whole-exome capture was relatively efficient, covering ~81% of the target region at minimal 20× coverage (Table 1). We detected an average of 69601.5 variants comprised of 61074.5 single nucleotide variants (SNVs) and 8777.4 indels per subject. All exome sequences reported in this study were deposited in SRA (https://submit.ncbi.nlm.nih.gov/subs/sra/). Accession codes for the exome-sequenced samples is reported in Supplementary Table 1.

**Table 1:** Overview of whole exome sequencing data.

	Average	Percent (%)
Total bases(bp)	8,481,712,437	100
Q30 bases(bp)	7,101,724,855	85.09
Total reads	72,234,480	100
Aligned paired reads	72,018,643	99.7
		Standard Deviation
Read depth	47.84	11.7078
1× coverage	0.9839	0.0009
10× coverage	0.9416	0.0400
20× coverage	0.8177	0.1057
30× coverage	0.6599	0.1415

### Identification and validation of the de novo mutations

Using the *PossibleDeNovo* module in GATK, we identified 33 putative DNMs from 51 SCZ trios. The newly identified DNMs included 2 uncategorized genetic mutations and 1 splice site mutation that produced a truncated protein (Supplementary Table 2). Next, we excluded the mutations in the two uncategorized genes (*C21orf2* and *C8orf48*) from the following validation. Direct PCR amplification and Sanger sequencing of each gene with candidate DNMs were performed on all 51 recruited SCZ trios. In total, 23 (74%) candidate mutations were validated as true DNMs in the SCZ probands. They were mapped to the 23 coding sequences (CDS) of the genes of 19 SCZ patients (Table 2). The nonsynonymous-to-synonymous (NS/S) mutation ratio was 3.2 and was very close to the NS/S ratio of 3.1 reported in an earlier SCZ study of DNMs using WES data (*n* = 617 SCZ trios, chi-square test *P* = 0.956). This previous report had also suggested that there was no significant elevation of DNMs in SCZ patients compared to neutral expectation (NS/S ratio = 2.8, *n* = 731 controls trios, chi-square test *P* = 0.797)^31^.

**Table 2:** List of the validated de novo mutations in Chinese trios.

Proband ID	Chromosome	Position	Gene Name	Transcript ID	Mutation type	Nucleotide change	AA change	Polyphen2_HVAR_score	Polyphen2_HVAR_pred
Sample_1079-3	chr17	42458249	*ITGA2B*	NM_000419	nonsynonymous	c.C1391T	p.P464L	1	D
Sample_1848-3	chr17	74944111	*MGAT5B*	NM_198955	nonsynonymous	c.G2150A	p.G717E	1	D
Sample_1874-3	chr21	32617835	*TIAM1*	NM_003253	nonsynonymous	c.G1553A	p.S518N	0.998	D
Sample_846-3	chr11	65486372	*KAT5*	NM_001206833	nonsynonymous	c.A1297G	p.K433E	0.95	D
Sample_1173-3	chr18	74154775	*ZNF516*	NM_014643	nonsynonymous	c.T236G	p.I79S	0.939	D
Sample_1097-3	chr11	47819409	*NUP160*	NM_015231	nonsynonymous	c.A3211G	p.R1071G	0.798	P
Sample_1049-3	chr1	223284633	*TLR5*	NM_003268	nonsynonymous	c.T1741C	p.F581L	0.584	P
Sample_1097-3	chr2	32449750	*NLRC4*	NM_001302504	nonsynonymous	c.C872A	p.A291D	0.398	B
Sample_689-3	chr21	34399472	*OLIG2*	NM_005806	nonsynonymous	c.T302C	p.M101T	0.295	B
Sample_1861-3	chr17	74533618	*CYGB*	NM_134268	nonsynonymous	c.A7G	p.K3E	0.124	B
Sample_841-3	chr2	73495972	*FBXO41*	NM_001080410	nonsynonymous	c.G787A	p.E263K	0.112	B
Sample_1874-3	chr19	54723009	*LILRB3*	NM_001081450	nonsynonymous	c.A1415G	p.H472R	0.032	B
Sample_1056-3	chr10	121658253	*SEC23IP*	NM_007190	nonsynonymous	c.C478G	p.P160A	0.009	B
Sample_841-3	chr2	128712785	*SAP130*	NM_024545	nonsynonymous	c.A2170G	p.I724V	0.007	B
Sample_1488-3	chr1	23763715	*ASAP3*	NM_001143778	nonsynonymous	c.A1223T	p.H408L	0	B
Sample_1128-3	chr4	52765485	*DCUN1D4*	NM_001040402	nonsynonymous	c.T556C	p.F186L	0	B
Sample_975-3	chr8	27507253	*SCARA3*	NM_016240	stop gain	c.C42A	p.C14X	.	.
Sample_641-3	chr14	92469762	*TRIP11*	NM_004239	splicing	.	.	.	.
Sample_549-3	chr14	73640385	*PSEN1*	NM_000021	synonymous	c.G450T	p.L150L	.	.
Sample_1067-3	chr8	59728197	*TOX*	NM_014729	synonymous	c.G1092A	p.Q364Q	.	.
Sample_769-3	chr7	107013182	*COG5*	NM_001161520	synonymous	c.T786G	p.A262A	.	.
Sample_939-3	chr9	99797910	*CTSV*	NM_001201575	synonymous	c.A687T	p.A229A	.	.
Sample_1173-3	chr17	40275186	*HSPB9*	NM_033194	synonymous	c.C318T	p.L106L	.	.

### Identification and validation of the compound heterozygous mutations

We identified 171 genes with rare protein-altering compound heterozygous mutations from 51 SCZ trios (3.35 genes per proband). On average of each proband, 3.26 genes with rare protein-altering compound heterozygous mutations were detected in 23 male probands and 4 genes with rare protein-altering compound heterozygous mutations were found in 24 female probands (without significant differences, two-tailed *t*-test *P* = 0.17). We found that the rare protein-altering compound heterozygous mutations in titin (*TTN*) gene were present in 14 trios. The result of standard Sanger sequencing of SCZ probands and parental DNA was used to validate 13 genes (including *TTN* gene), which were found to contain rare protein-altering compound heterozygous mutations in more than one trio (Supplementary Table 3). Rare protein-altering compound heterozygous mutations in 12 genes were eventually validated as true markers appearing in more than one trio. Only mucin 17 (*MUC17*) gene was found to be a false positive. In addition, considering the special profile of *TTN* gene (it is a very large gene, and there are often many unexplained variations in sequencing data analysis), we did not consider it a valuable candidate gene associated with SCZ.

### The transmission disequilibrium test (TDT)

**Fig. 2:**
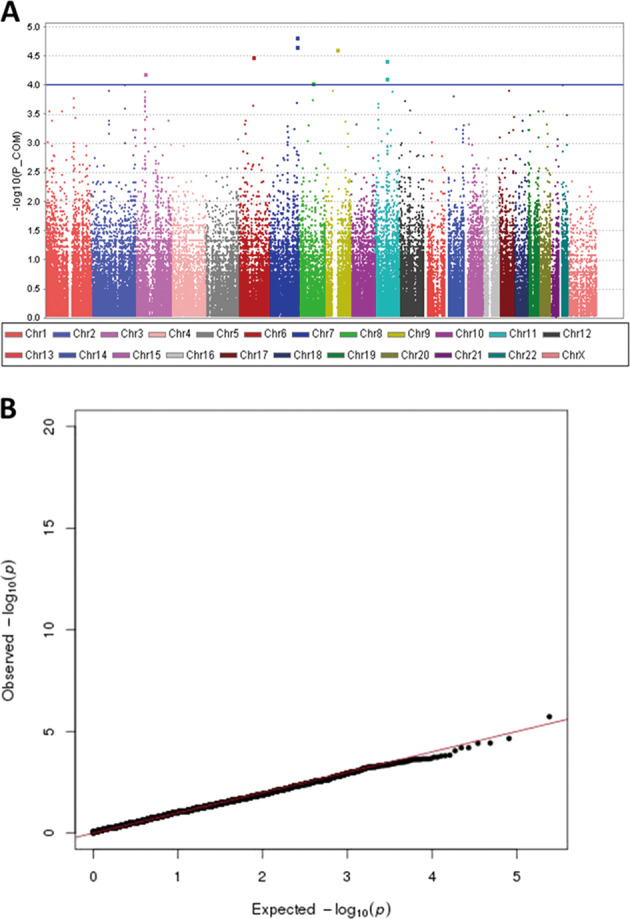
TDT analysis Manhattan and Q-Q plot. **a** We performed a transmission disequilibrium test (TDT) for 65 schizophrenia families. The blue line means *P*-value is 1 × 10^−4^. The most significant SNP is rs1051823, which was located in replication initiator 1 (*REPIN1*) gene downstream region (OR: 2.86; CI 95%: 1.59–5.16; *P*-value = 1.495 × 10^−5^). **b** This figure shows the expected distribution of *P*-values plotted against the observed distribution of combined asymptotic test *P*-value (because of the heterogeneity brought by the 14 SCZ families with affected parents and the impact of parental illness states on transmission disequilibrium) for our transmission disequilibrium test. Our study did not show any signs of global inflation of test statistics with 1.002.

TDT were performed using PLINK software on all 65 SCZ families to investigate the genetic associations in SCZ. A total of 136,894 SNPs remained after QC procedure and were included in the family-based association analyses. Combined asymptotic test *P* value was used as threshold of significance because of the heterogeneity brought by the 14 SCZ families with affected parents and the impact of parental illness states on transmission disequilibrium. A power calculation indicated that in 50 cases we had adequate power of 85% ~95% to detect many of the SNPs with medium effect size (larger than 0.15, Supplementary Fig. 1). Since the number of our families was not very large, we applied a stringent significance threshold at *P* of 5 × 10^−4^, which was most commonly used in similar studies (Fig. 2). This analysis revealed that there were 75 SNPs showed statistical significance in their associations with SCZ (Supplementary Table 4). In particular, the 26 exonic SNPs located on 18 candidate genes demonstrated significant signals. The highest associated SNP was rs1051823, which was located in replication initiator 1 (*REPIN1*) gene downstream region (*P* *=* 1.495 × 10^−5^; OR: 2.86; CI 95%: 1.59–5.16). In addition, 4 other SNPs with significant associations (rs1051764, rs1051760, rs3735165 and rs6722) were also found to be in the *REPIN1* gene. Finally, our results supported earlier discoveries of the important role of 3p21.1 loci in *NEK4-ITIH1-ITIH3-ITIH4* region in SCZ by finding a number of significant SNPs in this specific multigenetic region^41^. Another genetic association study also found 3p21.1 (including *PBRM1*, strong linkage disequilibrium made it difficult to pinpoint the risk genes) as risk loci for bipolar disorder (BD), SCZ and psychosis^42^. We extracted the 18 significant SNPs locating at 3q21.1 and made a linkage disequilibrium analysis. The results showed that they have a strong linkage relationship, as shown in Supplementary Fig. 2.

### Prediction for pathogenic potential of candidate genes

**Fig. 3:**
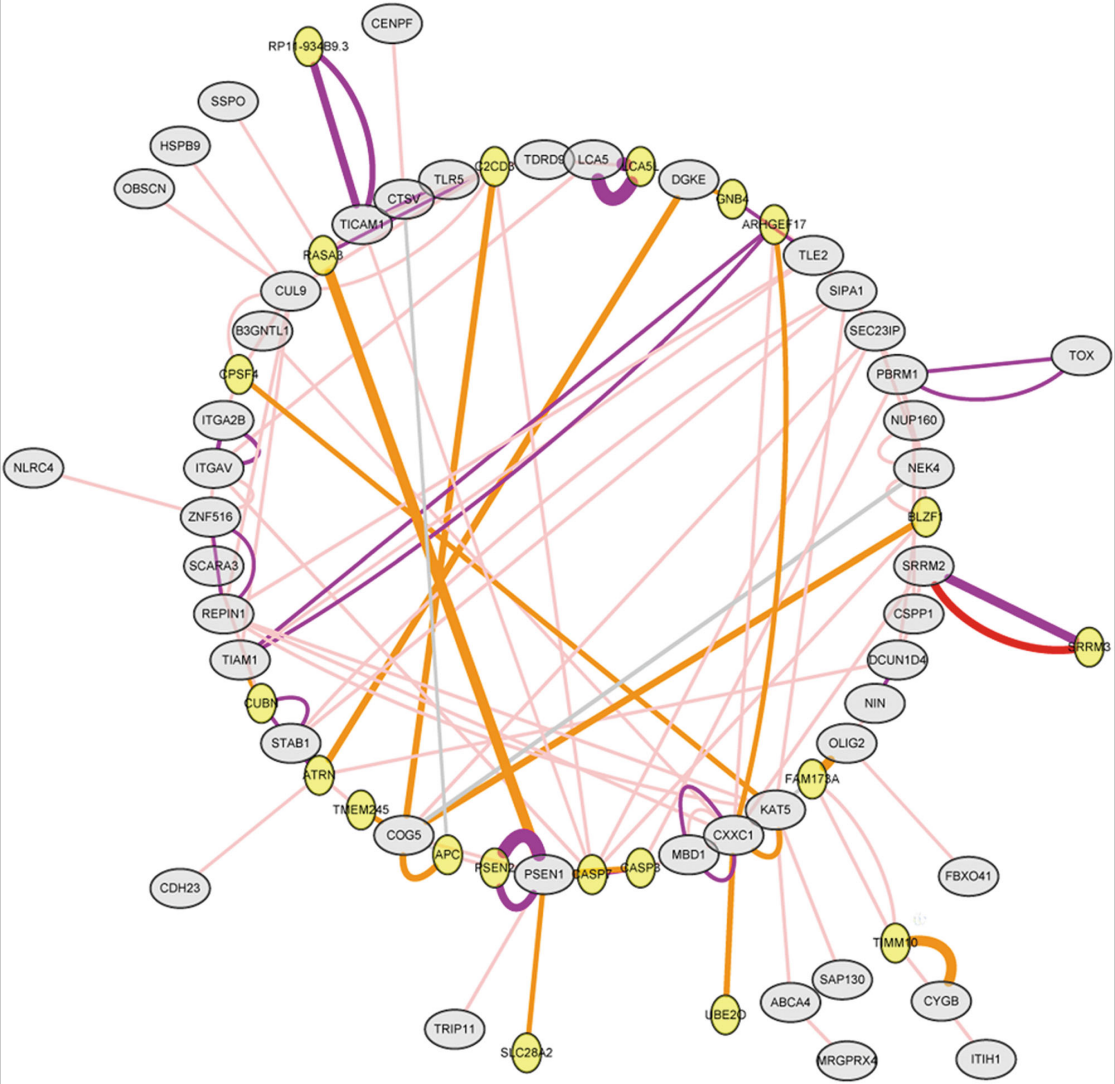
Biological network integration of candidate genes by GeneMania. Each node indicates a gene and each edge denotes an interaction between a pair of genes. Larger gray nodes represent the candidate genes entered, and smaller yellow nodes represent the derived interaction genes. A purple edge denotes a co-expression are reported in previous studies, gray edge denotes paired genes shared protein domains, pink denotes paired genes had physical interactions, whereas the orange edge denotes a predicted gene co-expression (PubMed: 12934013). The thickness of the edge indicates the weight in whole interaction network.

Firstly, we compared our results with earlier studies to explore the potential pathogenic effects of the 23 candidate genes that harbored the validated DNMs, 12 candidate genes with genuine rare protein-altering compound heterozygous mutations and 18 candidate genes with significant exonic SNPs identified by TDT analysis as described above. Several recent GWASs^14–16^ have shown that T-SNARE domain containing 1 (*TSNARE1*) and polybromo 1 (*PBRM1*) had a high correlation with SCZ. Previous genetic association studies^43,44^ have also revealed that oligodendrocyte transcription factor 2 (*OLIG2*) influences susceptibility to SCZ. Next, our results showed that *PBRM1* had the lowest RVIS score of −2.287 and a percentile of 2.23%, suggesting it is amongst the 2.23% most intolerant of human genes (Supplementary Table 5). Lastly, the output of GeneMANIA identified 123 interaction networks between the candidate genes (Supplementary Table 6), and the visualization of gene-gene interaction networks using Cytoscape software (Fig. 3). These interaction networks including co-expression are reported in previous studies, physical interactions from iRefIndex database and shared protein domains with interactions from Pfam or InterPro database. Among them, one co-expression interaction study surveyed the global expression patterns of 20 anatomically distinct sites of the human central nervous system^45^. We identified hub genes using cytoHubba, and validated the presenilin 1 (*PSEN1*) showing the most closely related to other nodes. In addition, 1 core module of this network for the candidate genes was structured, including genes: *KAT5*, *CXXC1*, *ARHGEF17*, *REPIN1*, *TIAM1*, *TLE2*.

## Discussion

Although we have been studying SCZ for decades and also made some progress, our understanding of the etiology of SCZ is still not very clear and an open question to be addressed. Accumulating data had indicated that genetic factors play a key role in SCZ mechanism, enabled by a large number of studies on genetic variations. Despite the significant increase in statistical power granted by these large-scale analyses, most existing genetic association studies only explained a fraction of SCZ onset or its heritability in families. Therefore, we took advantage of next-generation sequencing (NGS) technology and applied family-based WES to Chinese SCZ families in order to reveal the novel genetic associations to SCZ risk. Compared to traditional GWAS and candidate gene studies, WES is a more cost effective approach by having less bias and higher efficiency. In this study, we identified 23 DNMs and 12 genes with rare compound heterozygous mutations appearing more than one trio among the 51 SCZ trios that were with both healthy parents. It was noted that these 23 tag genes contained more nonsynonymous DNMs than synonymous DNMs. We also revealed 26 exonic or splice site SNPs showed statistical significance using TDT in all 65 SCZ families.

Studying DNMs is most effective in which the selective pressure is extremely strong and the effect size for those DNMs is large^46^. We found a de novo missense mutation p.G717E of mannosyl Alpha-1,6-Glycoprotein 6-Beta-N-Acetylglucosaminyltransferase B (*MGAT5B*) was identified in one SCZ family trio. This variant was estimated to be probably damaging, indicated by its Polyphen2 prediction score of 1. *MGAT5B* encodes a glycosyltransferases, which plays an important role in the nervous system by catalyzing the formation of a variety of glycoconjugates^47^. Toll like receptor 5 (*TLR5*) is the only previously reported gene that has been found to be associated with onset of SCZ in similar trio-based study using WES data in Bulgaria^31^. This gene encodes a member of the toll-like receptor (TLR) family, which plays a crucial role in pathogen recognition and activation of innate immune responses^48^. In addition, the relationship of neuronal and immunological pathways featured among top hits in recent psychiatric GWAS^49^, suggesting that *TLR5* we identified in this study may exert substantial influence on SCZ risk. At last, *OLIG2*, a gene known to be associated with SCZ susceptibility, has also been found to be a part of the genetic network underlying oligodendrocyte function in earlier investigations^43,44^.

It is also important to identify rare variations in SCZ patients, since they usually carry a large effect size^50^. In addition, the exploration of genes harboring rare missense mutations in different sporadic SCZ is an alternate way forward to resolve SCZ’s complexity^51^. Thus, we payed close attention to those genes that harboring rare compound heterozygous mutations in multiple trios. Our analysis of rare compound heterozygous mutations revealed that the genetic alterations in *TSNARE1*, SCO-Spondin (*SSPO*) genes may associated with onset of SCZ. The function of the *TSNARE1* gene is not well-understood, but a recent study suggested it may have a vertebrate-specific function in intracellular protein transport and synaptic vesicle exocytosis^52^. Moreover, a study showed that altering the expression of *TSNARE1* affects neurodevelopment in zebrafish^53^. The subcommissural organ (SCO) is a brain gland located in the roof of the third ventricle that releases glycoproteins into the cerebrospinal fluid, and there is evidence suggesting that serotonin (5HT) participates in the regulation of the SCO secretory activity in the rat^54,55^. Another study provided evidence of the statement that the behavioral data of processing speed revealed association with a locus that included *SSPO*^56^.

Furthermore, we made full use of the characteristics of the family through the TDT method to find the susceptibility gene of SCZ. In this way, we found one gene stabilin 1 (*STAB1*) might contribute to schizophrenia susceptibility previously identified by pathway analysis^57^. Another study suggested that *STAB1* is a new candidate gene for bipolar disorder combining of gene through expression and GWAS data^58^. Most noteworthy, we confirmed the importance of the *NEK4-ITIH1-ITIH3-ITIH4* region locating in 3p21.1 (including *PBRM1, STAB1*) in the pathogenesis of psychiatric disease^41,42,59^. The linkage disequilibrium analysis results showed that significant SNPs in this region have a strong linkage relationship. Inter-Alpha-Trypsin Inhibitor Heavy (ITIH) molecules belong to a family of serine protease inhibitors, and had been implicated in SCZ^60^, inflammatory diseases^61,62^ and carcinogenesis^63^.

In the silico analysis, we found *PBRM1* and *OLIG2* have been reported by the previous studies several times. And, the lowest RVIS score represented *PBRM1* was very stable among human genes. Thus the mutations found on this gene are likely to be an important risk site. In addition, these gene interaction networks linked related candidate genes together, and also found more genes that may be associated with schizophrenia based on existing candidate genes. Each pair of interaction proteins was calculated to pick out the hub gene and core module of the whole network. They provide the primary choice for future functional studies. Such as, the interaction network result suggested *PSEN1* was the most significant hub gene, and there have been reported *PSEN1* involved in the regulation of neurite outgrowth and Alzheimer’s disease^64^.

This study has several limitations that should be taken into consideration. Firstly, the use of small sample size in our analysis may have resulted in limited power to detect more signals from rare variants. Second, we did not use other algorithms to identify DNMs, which may miss some valuable findings. Lastly, we did not give a complete explanation of the interactions between the candidate genes and potential polygenic network underlying SCZ. Further functional studies may be helpful to make definitive conclusions of the associations and networks.

In summary, we provide a list of putative candidate genes (including *TSNARE1*, *PBRM1*, *OLIG2*, *STAB1*, *PSEN1*, *TLR5*, *MGAT5B* and *SSPO*) that were found to be associated with SCZ by performing WES on 65 Han Chinese SCZ families. Our findings of novel genetic markers will deepen the current knowledge of SCZ pathophysiology. In addition, we used a variety of strategies to comprehensively assess the genetic susceptibility to SCZ, supporting that SCZ does show strong heterogeneity and involve multiple genes and pathways. Further studies are warranted to validate our discoveries of the candidate gene markers and reveal their polygenic effects with other downstream target genes in the SCZ pathophysiology.

## Supplementary information

Supplementary Figures

Supplementary Table 1

Supplementary Table 2

Supplementary Table 3

Supplementary Table 4

Supplementary Table 5

Supplementary Table 6
