# Supplementary material for: Novel genetic susceptibility loci identified by family based whole exome sequencing in Han Chinese schizophrenia patients
Source: Transl Psychiatry. 2020 Jan 16;10:5. doi: 10.1038/s41398-020-0708-y (PMC7026419; doi:10.1038/s41398-020-0708-y)
Supplement: Supplementary file 1 — Supplementary Figures [file 41398_2020_708_MOESM1_ESM.docx]

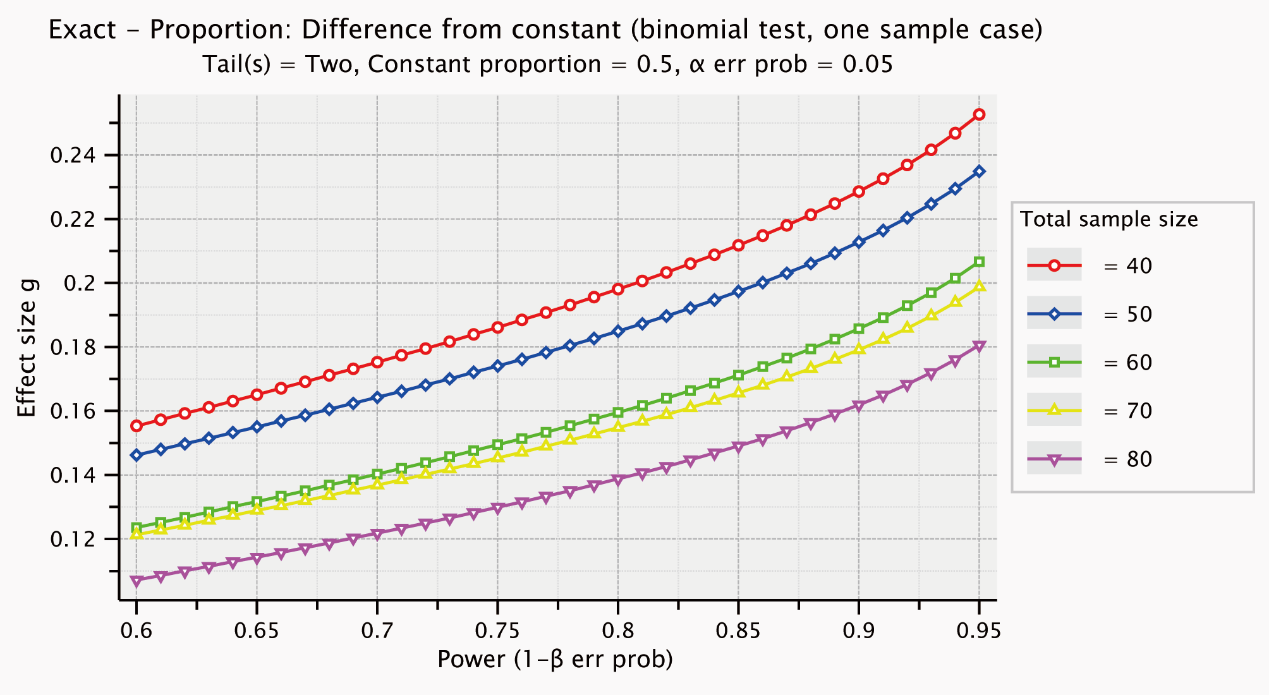


**Supplementary Figure 1 The calculation result of statistical power and effect size.** The family based transmission disequilibrium test (TDT) assumes that the allelic transmission is uniform, which means that the proportion is 0.5. We tested the statistical power and effect size for samples between 40 and 80.


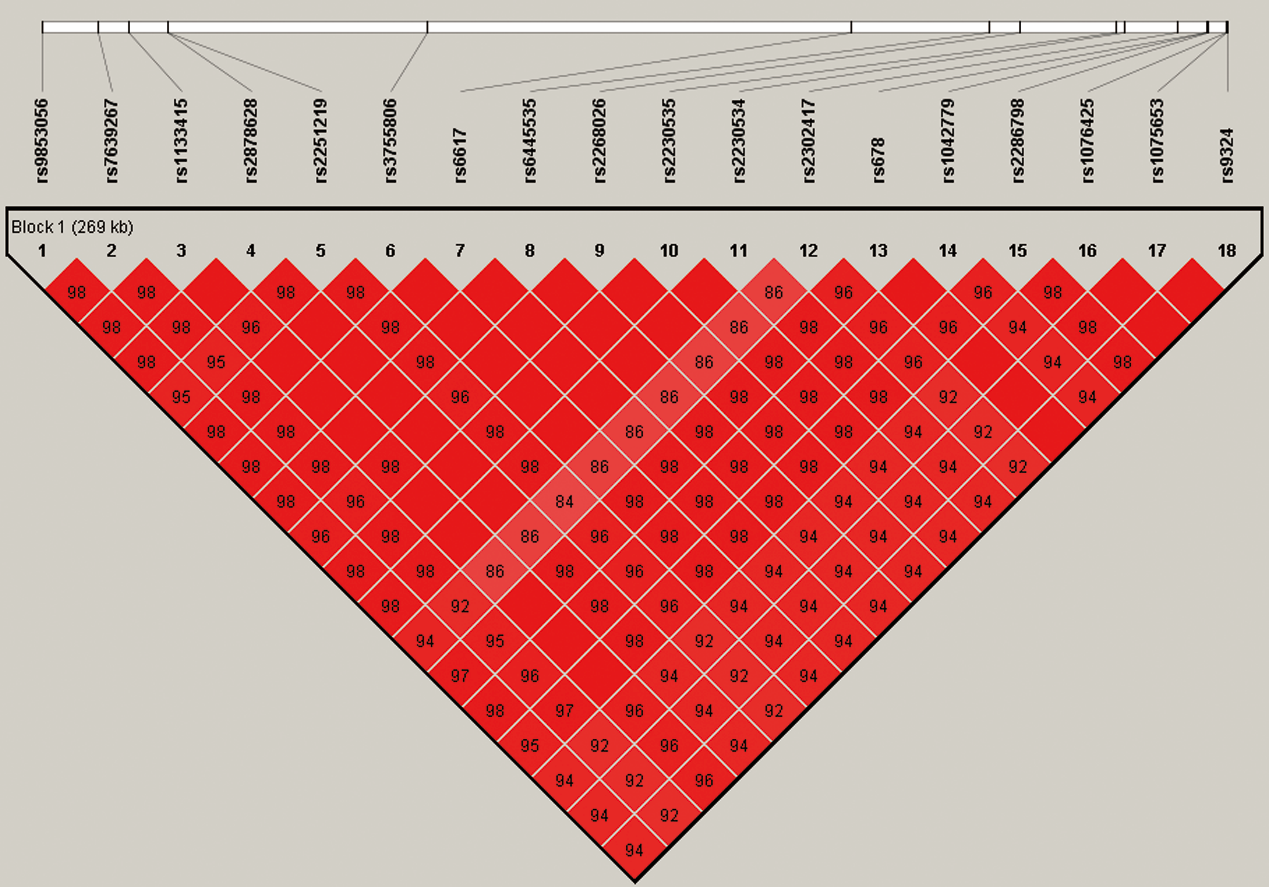


**Supplementary Figure 2 Linkage disequilibrium plot of significant** **SNPs locating at 3q21.1.** We use the D´parameter to determine the linkage disequilibrium between the two alleles. Color gradient from red to light red is correlated with D´value from high to low. The results showed that SNPs locating at 3q21.1 have a strong linkage relationship.
